# Video feedback to update negative self-perceptions in social anxiety disorder: A comparison of internet-delivered vs face-to-face cognitive therapy formats

**DOI:** 10.1016/j.jad.2023.03.017

**Published:** 2023-06-15

**Authors:** Jennifer Wild, Emma Warnock-Parkes, Richard Stott, Amy P.L. Kwok, Mandy H. Lissillour Chan, Candice L.Y.M. Powell, Patrick W.L. Leung, David M. Clark, Graham R. Thew

**Affiliations:** a Department of Experimental Psychology, University of Oxford, UK; b Phoenix Australia, Department of Psychiatry, University of Melbourne, Australia; c King's College London, London, UK; d Department of Clinical Psychology, Hong Kong East Cluster, Hospital Authority, Hong Kong, China; e New Life Psychiatric Rehabilitation Association, Hong Kong, China; f Mind HK, Hong Kong, China; g Department of Psychology, The Chinese University of Hong Kong, Hong Kong, China; h Oxford University Hospitals NHS Foundation Trust, UK; i Oxford Health NHS Foundation Trust, UK

**Keywords:** Social anxiety disorder, Cognitive behavioural therapy, Video feedback, Internet interventions, Self-perception, Remote therapy

## Abstract

**Background:**

Video feedback is a technique used in cognitive therapy for social anxiety disorder (CT-SAD) to update patients' negative self-perceptions of how they appear to others. Clients are supported to watch video of themselves engaging in social interactions. While typically undertaken in session with a therapist, this study aimed to investigate the effectiveness of remotely delivered video feedback embedded within an Internet-based cognitive therapy program (iCT-SAD).

**Methods:**

We examined patients' self-perceptions and social anxiety symptoms before and after video feedback in two randomised controlled trials. Study 1 compared 49 iCT-SAD participants with 47 from face-to-face CT-SAD. Study 2 was a replication using data from 38 iCT-SAD participants from Hong Kong.

**Results:**

In Study 1, ratings of self-perceptions and social anxiety showed significant reductions following video feedback, in both treatment formats. 92 % of participants in iCT-SAD, and 96 % in CT-SAD thought they looked less anxious compared to their predictions after viewing the videos. The change in self-perception ratings was larger in CT-SAD compared to iCT-SAD, but there was no evidence that the impact of video feedback on social anxiety symptoms around a week later differed between the two treatments. Study 2 replicated the iCT-SAD findings of Study 1.

**Limitations:**

The level of therapist support in iCT-SAD videofeedback varied with clinical need and was not measured.

**Conclusions:**

The findings indicate that video feedback can be delivered effectively online, and that its impact on social anxiety is not significantly different from in-person treatment delivery.

## Highlights

• Video feedback led to significant changes in self-perceptions and social anxiety.
• The impact on social anxiety did not significantly differ between therapy formats.
• 92 % and 96 % of participants in iCT-SAD and CT-SAD felt that they looked less anxious than predicted.

## 1 Introduction

Video feedback is used in cognitive therapy for social anxiety disorder (CT-SAD) to update patients' negative self-perceptions about how they appear to others. The technique involves the patient watching video of themselves engaged in a social interaction and comparing their predictions about how they think they will appear to others with how they objectively come across on video. Patients usually discover there is a discrepancy between how they think they come across with how they appear on video. Such learning is associated with significant reductions in social anxiety in the week after the procedure (McManus et al., 2009). Many studies have demonstrated the efficacy of video feedback when delivered in person (e.g., Harvey et al., 2000; Kim et al., 2002; Orr and Moscovitch, 2010; Parr and Cartwright-Hatton, 2009; Rapee and Hayman, 1996). In CT-SAD, video feedback is an interactive process. The therapist is on hand to set up the procedure, watch the video with the patient and address processing biases which could prevent patients from gaining corrective information while seeing themselves on camera. In recent years CT-SAD, which is recommended as a first-line treatment for SAD by the UK's National Institute for Health and Care Excellence (NICE, 2013), has been translated into a therapist-assisted internet-delivered format (iCT-SAD). In iCT-SAD, the treatment procedures, including video feedback, are delivered in an internet program (see Stott et al., 2013 for more details). While the two treatment formats achieve similar overall outcomes (Clark et al., 2022), it is unclear whether specific procedures within the treatment achieve similar results in each format.

One might think that video feedback would be more efficacious in face-to-face CT-SAD compared to iCT-SAD since the therapist has an active role in the delivery of video feedback in face-to-face treatment. The therapist sets up the procedure, guides the patient on how to watch the video, and addresses processing biases which could interfere with the procedure achieving maximum benefit. For example, the therapist will guide the patient to observe the video of themselves as though they are observing a stranger, ignoring their feelings and only focusing on what they can see and hear on screen in order to prevent patients from re-experiencing anxiety when watching video of themselves. The therapist may pause the video and ask the patient to rate how self or externally focused they are to gauge whether they are observing the video objectively. They may then pose questions in order to facilitate a more externally focused view of the video. The therapist will help patients to overcome the tendency to criticise their performance when watching the video by giving them written feedback from their conversational partner before watching the video with the aim of eliciting a kinder mode of viewing. The therapist will address a range of processing biases when delivering video feedback in person; for a fuller discussion, see Warnock-Parkes et al. (2017). In iCT-SAD, a specialized module guides patients to prepare for viewing the video in a similar way to face-to-face therapy but the therapist is not on hand to intervene to address processing biases which could arise while patients are watching video of themselves. Thus, it would not be surprising if video feedback delivered during a course of iCT-SAD may be less effective than when delivered during CT-SAD. Comparing the delivery formats of video feedback could offer valuable guidance for improvements in the procedure.

While there are many programs for SAD that will introduce feedback in different ways and at different stages of therapy, during a course of CT-SAD, video feedback is introduced for the first time in a standardised way in the third session when patients will experience video feedback of the self-focused attention and safety behaviours experiment.^1^ Patients will watch video of two social interactions they had in session two of their treatment. In the first conversation, they had been instructed to adopt self-focused attention and to use their safety behaviours, such as censoring what to say, covering a blush and preparing topics in advance. In the second conversation, they have been instructed to adopt externally focused attention and to drop their safety behaviours. Before viewing the conversations on video, the patient will make predictions about the visibility of their feared outcomes, anxious appearance and overall performance. In CT-SAD, the patient will watch the video of themselves and guided by their therapist, will re-rate their predictions during the course of a 90 mins session.

When working with patients receiving iCT-SAD, the therapist has a much less active role. On average during the 14-week treatment, the therapist will deliver 4.1 h of live contact compared with 18.4 h in CT-SAD (Clark et al., 2022). The majority of therapist-patient communication in iCT-SAD is asynchronous meaning the majority of contact is through messaging. Patients complete the self-focused attention and safety behaviours experiment via the webcam within the program in the second week of treatment. They then complete a module which guides them to view the video of the experiment. It is unclear if video feedback can be delivered effectively in the absence of a therapist as is delivered in iCT-SAD. In this study, we compare patients' ratings of self-perceptions and symptoms of social anxiety before and after video feedback for patients who received the intervention as part of a randomised controlled trial of CT-SAD and for patients who received video feedback as part of RCTs of iCT-SAD in the UK and in Hong Kong. We hypothesise that video feedback delivered as part of iCT-SAD will evidence efficacy, and we explore how outcomes compare between the two therapy formats.

## 2 Study 1

### 2.1 Method

#### 2.1.1 Participants

This study analysed data from a UK randomised controlled trial in which participants were randomised to iCT-SAD, face-to-face CT-SAD, or waitlist (Clark et al., 2022). The study was prospectively registered (ISRCTN95458747) and received ethical approval. After the wait period, participants in this group were randomly allocated to one of the two treatment conditions. In total, 49 participants completed iCT-SAD, and 50 completed face-to-face CT-SAD. Both treatments showed large effect sizes for the reduction in social anxiety symptoms compared to waitlist, and scores on the primary outcome measure were not significantly different between the two. Video feedback data was available for all participants in the iCT-SAD group, and for 47 participants in the CT-SAD group (two participants did not complete the video feedback intervention, and for one participant these data were not available), giving a total of 96 participants in the present study. All were adults with a main diagnosis of SAD. In the iCT-SAD group, 26 participants were female, and the mean age was 31.9 (SD = 8.0). In the CT-SAD group, 23 participants were female, and the mean age was 32.1 (SD = 8.8).

#### 2.1.2 Measures

##### 2.1.2.1 Self-perception

Prior to watching the videos, participants were asked to rate on 0–100 scales: (a) how anxious do you think you will look?, (b) the extent to which they believe their idiosyncratic feared social outcomes will be evident (for example, how much do you think you will run out of things to say?), and (c) how well do you think you will perform overall? The same ratings were taken again immediately after watching each video. Mean ratings of anxiety, feared beliefs, and performance for each participant were taken by averaging across the two videos.

##### 2.1.2.2 Social anxiety

Social anxiety was measured using the self-report version of the Leibowitz Social Anxiety Scale (LSAS; Baker et al., 2002), a widely used measure of social anxiety symptoms that have occurred over the past week and which demonstrates good psychometric properties. This was completed by participants on a weekly basis in both CT-SAD and iCT-SAD. Unlike the measure of self-perception which could be administered immediately before and after video feedback, the LSAS could not be administered more frequently than weekly since it asks about symptom occurrence in the past week. The two LSAS scores either side of the video feedback intervention were used for analysis.

#### 2.1.3 Analysis

Comparisons of pre-video ratings between CT-SAD and iCT-SAD were performed using independent samples *t*-tests. Changes in ratings over time used paired samples t-tests, or in one instance where nonparametric assumptions were not met (violation of the normality assumption based on the Shapiro-Wilk test), a Wilcoxon signed rank test was used. Analysis of the change in ratings between CT-SAD and iCT-SAD was performed using linear mixed effects models. These models included categorical fixed factors of time (pre-video versus post-video) and condition (CT-SAD versus iCT-SAD), and the time-by-condition interaction (to allow estimation of the condition effect at each timepoint). A random effect of participant was specified to account for between-subject variation. Models used restricted maximum likelihood estimation. The normality of residuals assumption was examined using Q-Q plots and was met for all models. All analyses were performed in R version 4.0.3 (R Core Team, 2020) including the package ‘nlme’ (Pinheiro et al., 2018).

### 2.2 Results

We first examined whether the pre-video ratings differed between CT-SAD and iCT-SAD. Independent samples *t*-tests indicated that the pre-video ratings of looking anxious (*t* (92) = −0.77, *p* = .441) and performance (*t* (76) = −0.79, *p* = .433) were not significantly different between the two treatment conditions. Pre-video ratings of feared beliefs were significantly higher in iCT-SAD compared to CT-SAD: *t* (94) = −2.01, *p* = .048, *d* = 0.41. Social anxiety scores on the LSAS prior to the video feedback intervention were not significantly different between conditions: *t* (94) = 0.22, *p* = .830.

Table 1 shows the mean ratings given by participants before and after video feedback. Results of paired *t*-tests indicated that after viewing themselves on video, there were significant decreases in participants' ratings of how anxious they looked, and the extent to which their feared beliefs occurred, as well as a significant increase in the rating of their overall performance. Scores on the LSAS also showed a significant decrease suggesting a drop in social anxiety in the week following the video feedback intervention. This pattern of results was observed for both CT-SAD and iCT-SAD.

**Table 1:** Comparison of participants' ratings before and after video feedback in iCT-SAD and CT-SAD (Study 1).

Treatment	Measure	N	Before video mean (SD)	After video mean (SD)	Mean difference [95 % CI]	Test statistic	*p*	Cohen's *d*
iCT-SAD	Look anxious (0–100)	49	57.70 (15.38)	33.21 (20.98)	24.49 [19.16–29.82]	9.24	<.001	1.59
	Mean social fear belief (0–100)	49	53.92 (14.82)	28.46 (14.41)	25.46 [21.72–29.19]	13.70	<.001	1.72
	Overall performance (0–100)	49	50.71 (12.92)	63.32 (13.94)	13.75 [10.00–17.50]	W = 77.50	<.001	0.98
	LSAS	49	72.61 (17.94)	64.98 (20.64)	7.63 [4.20–11.07]	4.47	<.001	0.43
CT-SAD	Look anxious (0–100)	45	55.14 (16.73)	22.89 (18.35)	32.24 [26.37–38.11]	11.07	<.001	1.93
	Mean social fear belief (0–100)	47	47.99 (14.12)	14.60 (14.60)	33.39 [29.22–37.56]	16.12	<.001	2.36
	Overall performance (0–100)	29	53.16 (13.75)	75.91 (14.65)	22.76 [17.90–27.62]	−9.60	<.001	1.66
	LSAS	47	73.47 (20.85)	65.45 (22.74)	8.02 [4.26–11.78]	4.29	<.001	0.38

Notes. Only a subset of participants were asked to rate their overall performance by their therapists in face-to-face CT-SAD. LSAS = Liebowitz Social Anxiety Scale. Test statistic is *t* unless indicated. W = Wilcoxon signed rank test. Cohen's d calculated as Before video mean − After video mean / baseline standard deviation.

Results of the linear mixed effects models indicated that for ratings of looking anxious, there was a significant adjusted group difference at the post-video timepoint, indicating greater change over time in CT-SAD compared to iCT-SAD (estimate = −10.32, *SE* = 3.72, *p* = .007). The same was true for ratings of feared beliefs (estimate = −13.86, *SE* = 2.96, *p* < .001), and for ratings of overall performance (estimate = 12.60, *SE* = 3.22, *p* < .001). However, for scores on the LSAS the adjusted group difference at the post-video timepoint was not significant (estimate = 0.47, *SE* = 4.20, *p* = .912). Overall, 92 % of participants in iCT-SAD, and 96 % of participants in CT-SAD showed a decrease in their rating of how anxious they thought they looked as a result of the video feedback intervention. All participants (100 %) in both treatments showed a decrease in their ratings of their feared beliefs, indicating an improvement in their self-perception compared to their predictions. Lastly, 88 % of participants in iCT-SAD, and 98 % of participants in CT-SAD, showed an increase in the ratings of their overall performance.

## 3 Study 2

### 3.1 Method

#### 3.1.1 Participants

Study 2 analysed data from a randomised controlled trial of iCT-SAD compared to waitlist undertaken in Hong Kong (Thew et al., 2022). Hong Kong provided an opportunity to test the iCT-SAD program in a contrasting cultural setting compared to the UK, with significant demand for psychological therapies and a sufficiently large English-speaking population to allow the program to be implemented without translation in this initial stage. Forty-four participants took part in this trial, of whom 38 completed the video feedback intervention in iCT-SAD and were therefore analysed in Study 2 (one participant withdrew from the waitlist condition; two declined to start treatment after completing the wait period; two withdrew from treatment prior to video feedback; and for one participant there was a technical error with the video recordings). All participants were of Chinese ethnicity, were resident in Hong Kong, and had sufficient English language skills to take part in the program, as determined through discussion during study recruitment. The inclusion and exclusion criteria for this study were the same as for the UK trial analysed in Study 1. Twenty-six of the 38 participants were female, and the mean age was 32.1 (SD = 9.4).

#### 3.1.2 Procedure and measures

The iCT-SAD video feedback procedure, and study measures, were identical to Study 1. The iCT-SAD program was administered in English for this study.

### 3.2 Results

The mean ratings given by participants before and after video feedback are shown in Table 2. As in Study 1, results of paired *t*-tests indicated that after viewing themselves on video, there were significant decreases in participants' ratings of how anxious they looked, and the extent to which their feared beliefs occurred, as well as a significant increase in the rating of their overall performance. 92 % of participants in iCT-SAD showed a decrease in their rating of how anxious they looked, 95 % showed a decrease in ratings of feared beliefs, and 84 % showed an increase in ratings of their overall performance as a result of the video feedback intervention.

**Table 2:** Comparison of participants' ratings before and after video feedback in iCT-SAD (Study 2).

Treatment	Measure	N	Before video mean (SD)	After video mean (SD)	Mean difference [95 % CI]	Test statistic	*p*	Cohen's *d*
iCT-SAD	Look anxious (0–100)	38	53.75 (18.15)	34.71 (16.22)	19.04 [14.41–23.67]	8.33	<.001	1.05
	Mean social fear belief (0–100)	38	41.36 (19.35)	23.02 (16.60)	18.34 [13.58–23.11]	7.80	<.001	0.95
	Overall performance (0–100)	38	53.42 (14.21)	63.78 (12.01)	12.50 [7.50–15.00]	W = 69.50	<.001	0.73
	LSAS	38	72.97 (23.47)	64.21 (25.08)	10.00 [3.00–15.00]	W = 451.00	.009	0.37

Notes. LSAS = Liebowitz Social Anxiety Scale. Test statistic is *t* unless indicated. W = Wilcoxon signed rank test. Cohen's d calculated as Before video mean − After video mean / baseline standard deviation.

To assess whether the iCT-SAD results from Study 2 were different to those of Study 1, we used linear mixed effects models to compare the extent of pre-post changes between Study 1 (UK) and Study 2 (Hong Kong). Models therefore used ‘study’ (UK versus Hong Kong) in place of ‘condition’ but were otherwise specified identically to Study 1. For ratings of looking anxious, the adjusted group difference at the post-video timepoint was not significant (estimate = 1.50, *SE* = 3.87, *p* = .700) indicating that the two studies did not significantly differ. For feared beliefs, pre-video ratings in the UK iCT-SAD sample were significantly higher than the Hong Kong iCT-SAD sample (estimate = −12.56, *SE* = 3.50, *p* = .001), but at the post-video timepoint the extent of change over time did not significantly differ between the two studies (estimate = −5.44, *SE* = 3.50, *p* = .124). For ratings of overall performance, the adjusted group difference at the post-video timepoint was not significant (estimate = 0.46, *SE* = 2.88, *p* = .873). The same was true for LSAS scores (estimate = −0.77, *SE* = 4.68, *p* = .870). Overall, the Study 2 findings therefore replicated those of Study 1.

## 4 Discussion

### 4.1 Study 1: can we change self-perceptions online as well as we can in face-to-face cognitive therapy?

The results of study 1 show that video feedback may be an effective intervention for changing negative self-perceptions when delivered face-to-face and as part of internet-delivered CT-SAD. Effect sizes for the change in negative self-perceptions after video feedback were large for both face-to-face and internet delivery. Results indicated that participants who received face-to-face CT-SAD showed more change in their negative self-perceptions after video feedback than in iCT-SAD. However, there was comparable improvement in subsequent social anxiety across both treatments. Furthermore, in the present study 92 % of participants who had iCT-SAD thought they looked less anxious following video feedback, compared to 96 % of patients in face-to-face delivery. This provides further evidence of the consistency of effects of video feedback in updating the negative self-perception that is central to SAD.

When we consider differences in the way video feedback is conducted face-to-face versus online, it is possibly not surprising that face-to-face delivery shows a larger change in negative self-perceptions. Patients with SAD are prone to a range of processing biases that can interfere when they view themselves on video. To maximise the effectiveness of video feedback it is key that patients are cognitively prepared to view the video, for example by making careful predictions in advance about what they think they will see based on their feelings and self-image (Harvey et al., 2000). It is also crucial that patients focus externally when viewing footage, ignoring their feelings or any self-critical thoughts that might interfere with the way they process the footage. In face-to-face treatment the therapist guides patients through this process live while viewing the video. The video is paused and discussed when needed to help overcome any processing biases and maximise the discrepancy between what the patient predicted they would see and what they perceive after viewing. This may provide more opportunity for spotting and addressing processing biases as they come up. In contrast, in online delivery a treatment module prepares patients to view themselves in an objective and non-critical mode (e.g., imagining they are viewing a stranger, looking at all the people on the screen etc.). A troubleshooting section then encourages patients who may have become self-critical when viewing, or used their feelings to judge what they saw, to view the video again in a less critical manner. Patients may have some discussion about their ratings with their therapist, either via the messaging system or during a phone call, but this is unlikely to be done in synchrony with viewing the video.

Despite differences in ratings of negative self-perceptions between the two methods of delivery, we observed no significant difference in improvement in weekly social anxiety scores assessed just before the next session with the therapist. It is possible that this might relate to the way in which ratings of self-perceptions following video feedback were recorded. In face-to-face delivery the final video feedback ratings were taken after a detailed therapist-patient discussion following viewing of the videos. In online treatment patients made their ratings immediately after watching the videos online and before any further discussion with their therapist that may have generated further, unrecorded improvement in self-perception. Some patients may even have viewed their videos again with the support of their therapist on the phone.

Alternatively, differences in the self-perception ratings observed between online and face-to-face delivery may be in part explained by what the patient is able to view on the video recordings. In face-to-face therapy patients see a recording of themselves speaking with their conversational partner in the same room. They are usually able to see both their whole body and that of their conversational partner. This may mean they can view a range of non-verbal cues (e.g., their conversational partner's hand gestures, open body posture etc.) that might not always be visible in an online video chat (see Grondin et al., 2019). Therefore, some non-verbal cues demonstrating that the person they are speaking to finds them acceptable could be missing in a video chat recording. It is also possible that viewing their face front-on in a video chat, rather than side-to-side in a face-to-face interaction, may make it more difficult for somebody with social anxiety to switch off from a self-critical mode when viewing video. Nevertheless, the comparable reduction in social anxiety scores after both online and face-to-face video feedback indicates that any limitations of a web-chat recording do not impact on the effectiveness of the intervention overall.

The finding that there was no difference between the two treatment formats in social anxiety scores following video feedback, despite there being a difference in ratings of self-perception after viewing the videos, could also in part be explained by the timing of these ratings. Ratings of self-perception were made immediately after watching the videos, but ratings of social anxiety on the LSAS were made sometime in the week following video feedback. In that week some iCT-SAD patients will have had access to a further two online modules: a module with online exercises to practice attention training (‘Getting out of your head and into the world’) and a module introducing the idea of behavioural experiments and encouraging patients to start planning and carrying out experiments (‘Behavioural Experiments’). These are additional interventions that patients in face-to-face treatment will have had much less access to in the post video feedback week.

One potential limitation is that there is some variation as to when patients completed the LSAS after their session on video feedback. For the most part patients in both CT and ICT will have completed the measure just before the start of the video feedback session and then the post measure was given no less than one week after the video feedback session. However, there was some small variability in the number of days after video feedback this would have been completed.

### 4.2 Study 2: replicating the effects of video feedback delivered through iCT-SAD in a Hong Kong sample

Study 2 demonstrates a replication of the effects of video feedback when embedded within the iCT-SAD program in a Hong Kong sample. As found in Study 1, after patients viewed themselves on video through the online program, there were significant decreases in how anxious patients thought they looked and the extent to which they believed their fears happened, with 92 % of patients receiving iCT-SAD showing a decrease in how anxious they thought they looked after video feedback. There was a significant improvement in patients' perceptions of their overall social performance. This provides further evidence, from a sample from a different cultural background, that video feedback shows consistently positive effects on SAD and can be effectively delivered online as part of iCT-SAD.

### 4.3 Summary and concluding discussion

Study 1 and Study 2 suggest that video feedback, conducted via internet-delivered CT-SAD, may be an effective intervention for changing negative self-perceptions and is followed by significant reduction in social anxiety when embedded within a series of online modules. The present paper confirms findings reported by McManus et al. (2009) and Warnock-Parkes et al. (2017) that video feedback appears to be an effective intervention in the treatment of social anxiety. The study extends these findings to demonstrate that it can be delivered remotely, and without the presence of a therapist, through an online treatment program (iCT-SAD). Previous studies of iCT-SAD provided preliminary evidence that video feedback could be delivered online (Clark et al., 2022; Stott et al., 2013; Thew et al., 2019, Thew et al., 2022), but this is the first study suggesting the effectiveness of video feedback specifically.

Video feedback works by updating the distorted image or impression patients have of themselves in social situations that maintain SAD (Clark and Wells, 1995; Hirsch et al., 2004). The improvement in social anxiety scores observed following video feedback could be explained by the theory that patients who have a better social image or impression of themselves experience less social anxiety and avoidance. This is the first paper of its kind to demonstrate this effect following video feedback from a video call interaction, indicating that learning from behavioural experiments conducted via a video link generalises to face-to-face social situations.

A limitation of these studies is that in previous papers on the effectiveness of video feedback in face-to-face treatment (McManus et al., 2009; Warnock-Parkes et al., 2017) we were able to demonstrate the differential effects of video feedback over and above previous sessions (e.g. developing the model and the self-focused attention and safety behaviours experiment) as each procedure was delivered in a different session. However, in online delivery a number of patients will have completed all interventions in the same week and so we are unable to demonstrate the individual effects of video feedback in the same way.

No measure was given of how much therapists intervened or helped guide patients during the UK or Hong Kong studies of internet delivered video feedback. For example, some patients may have needed additional help during a telephone call session to complete video feedback. It is therefore difficult to conclude what level of independence patients need when doing video feedback to achieve significant effects. Further research could investigate whether additional therapist input around video feedback online has any impact on its effectiveness. Finally, we were unable to include our waitlist conditions in analyses since patients allocated to waitlist in each study completed measures at baseline, mid and post-wait-list rather than weekly (Clark et al., 2022; Thew et al., 2022). It is possible that the improvements in self-perceptions and social anxiety symptoms observed with video feedback may be due to or influenced by repeated measurements. However, we believe this is unlikely, since our waitlist conditions showed no improvements on measures of social anxiety at any time point (Clark et al., 2022; Thew et al., 2022), indicating that repeated measurement is unlikely to account for the improvements that appear to be associated with video feedback.

Internet delivered treatments have the potential to provide effective, accessible, low-cost therapy for patients with SAD that save on therapist time (Stott et al., 2013; Thew et al., 2019). A challenge for online treatments is whether key therapeutic manoeuvres, such as video feedback, that update patient's negative self-images, can be delivered over the internet. This paper is the first of its kind to demonstrate that not only can video feedback be delivered online, but that the associated reduction in social anxiety is comparable to when delivered in person.

## Funding

JW and DMC are supported by the 10.13039/100010269Wellcome Trust [069777, 200796, 00070]. GRT is supported by the 10.13039/100010269Wellcome Trust [102176], the 10.13039/501100013373NIHR Oxford Health Biomedical Research Centre, and the 10.13039/100014461NIHR Oxford Biomedical Research Centre. The views expressed are those of the authors and not necessarily those of the NHS, the NIHR or the Department of Health.

## CRediT authorship contribution statement

JW, EWP, GT, DMC: conceptualisation, investigation, methodology. JW, EWP, GT: writing original draft; DMC: review and editing. JW, EWP, RS, APLK, MHLC, CLYMP, PWLL: investigation. GT, DMC data curation, formal analysis. DMC, GT, JW: funding acquisition. DMC, JW, GT: supervision.

## Conflict of interest

The authors declare no conflict of interest.

## Footnotes

1 For a description of this experiment and other CT-SAD treatment components, please see (Clark, 2005), Warnock-Parkes et al. (2020) and the Oxford Centre for Anxiety Disorders and Trauma free resources website, www.oxcadatresources.com.
